# Supplementary material for: Prevalence of Vitamin B12 and Folate Deficiencies in Indian Children and Adolescents
Source: Nutrients. 2023 Jul 3;15(13):3026. doi: 10.3390/nu15133026 (PMC10346745; doi:10.3390/nu15133026)
Supplement: Supplementary file 1 [file nutrients-15-03026-s001.zip › nutrients-2423520-supplementary.pdf]

Prevalence of Vitamin B_{12} and Folate Deficiencies in Indian Children and Adolescents

Tattari Shalini [1], Raghu Pullakhandam [1], Santu Ghosh [2], Bharati Kulkarni [1], Hemalatha Rajkumar [1], Harshpal S. Sachdev [3], Anura V. Kurpad [2] and Geereddy Bhanuprakash Reddy [1,*]

[1] National Institute of Nutrition, Jamai-Osmania, Tarnaka, Hyderabad 500007, India
[2] St. John's Medical College, Bangalore 560034, India; santu.g@stjohns.in (S.G.)
[3] Sitaram Bhartia Institute of Science and Research, New Delhi 110016, India
* Correspondence: geereddy@yahoo.com or reddyg.bp@icmr.gov.in; Tel.: +91-40-27197252

Running title: Vitamin B_{12} and folate status in Indian children and adolescents

Supplementary Table S1. Comparison of characteristics of the study (vitamin B12 and folate) sample with the total survey sample.

Characteristics		1-4 years			5-9 years			10-19 years		
		Vitamin B12 % (95% CI)	Folate % (95% CI)	CNNS total % (95% CI)	Vitamin B12 % (95% CI)	Folate % (95% CI)	CNNS total % (95% CI)	Vitamin B12 % (95% CI)	Folate % (95% CI)	CNNS total % (95% CI)
Age	1-2 years	38.6 (36.4-40.9)	38.7 (36.7-40.7)	48.6 (47.5-49.6)	5-6 years: 39.7 (38.0-41.4)	38.8 (37.3-40.3)	40.4 (39.4-41.4)	10-14 years: 52.8 (51.4-54.3)	52.6 (51.3-53.9)	51.8 (51.2-52.5)
	3-4 years	61.4 (59.1-63.6)	61.3 (59.3-63.3)	51.4 (50.4-52.5)	7-9 years: 60.3 (58.6-62.0)	61.2 (59.7-62.7)	59.6 (58.6-60.6)	15-19 years: 47.2 (45.7-48.6)	47.4 (46.1-48.7)	48.2 (47.5-48.8)
Sex	Boys	52.9 (50.3-55.4)	52.5 (50.0-55.0)	51.4 (50.3-52.6)	51.6 (49.8-53.3)	51.2 (49.4-52.9)	50.4 (49.4-51.4)	50.6 (48.6-52.5)	50.6 (48.8-52.4)	49.9 (48.8-50.9)
	Girls	47.1 (44.6-49.7)	47.5 (45.0-50.0)	48.6 (47.4-49.7)	48.4 (46.7-50.2)	48.8 (47.1-50.6)	49.6 (48.6-50.6)	49.4 (47.5-51.4)	49.4 (47.6-51.2)	50.1 (49.1-51.2)
Residence	Urban	24.9 (21.7-28.4)	25.7 (22.5-29.1)	23.8 (21.4-26.3)	23.6 (20.7-26.8)	25.1 (22.1-28.2)	24.2 (21.8-26.8)	25.2 (22.1-28.6)	25.7 (22.6-29.0)	24.7 (22.3-27.4)
	Rural	75.1 (71.6-78.3)	74.3 (70.9-77.5)	76.2 (73.7-78.6)	76.4 (73.2-79.3)	74.9 (71.8-77.9)	75.8 (73·2-78·2)	74.8 (71.4-77.9)	74.3 (71.0-77.4)	75.3 (72.6-77.7)
Mothers' Schooling	Primary	34.3 (31.7-36.9)	34.6 (31.9-37.3)	37.8 (35.9-39.9)	47.8 (45.4-50.3)	47.9 (45.6-50.3)	49.3 (47.4-51.2)	16.3 (14.3-18.4)	15.9 (14.1-18.0)	14.6 (13.5-15.9)
	Secondary	44.3 (41.8-46.8)	43.6 (41.3-46.0)	41.8 (40.3-43.3)	40.1 (38.0-42.3)	39.8 (37.8-41.8)	37.9 (36.5-39.3)	68.7 (66.2-71.1)	69.3 (67.0-71.5)	69.1 (67.7-70.4)
	Higher Secondary	10.8 (9.3-12.5)	11.4 (9.9-13.1)	10.0 (9.2-10.9)	6.7 (6.0-7.6)	6.9 (6.2-7.8)	6.7 (6.2-7.3)	9.7 (7.7-12.1)	9.5 (7.7-11.6)	9.1 (8.4-9.9)
	Graduation & above	10.7 (9.2-12.4)	10.4 (9.0-12.0)	10.3 (9.5-11.2)	5.3 (4.7-6.1)	5.4 (4.8-6.1)	6.1 (5.5-6.6)	5.3 (4.4-6.3)	5.3 (4.5-6.3)	7.2 (6.5-8.0)
Wealth Index	Poorest	15.9 (13.8-18.3)	15.8 (13.7-18.1)	20.3 (18.1-22.6)	18.1 (16.2-20.3)	18.0 (15.8-20.4)	20.0 (18.0-22.2)	17.8 (15.5-20.4)	18.3 (16.0-20.8)	20.0 (17.9-22.3)
	Poor	21.2 (18.5-24.1)	20.5 (17.9-23.4)	19.4 (18.1-20.8)	21.3 (19.4-23.3)	20.2 (18.5-21.9)	20.0 (18.6-21.4)	20.5 (18.8-22.3)	20.6 (18.9-22.3)	20.0 (18.8-21.2)
	Middle	22.3 (20.4-24.3)	21.7 (19.9-23.5)	20.2 (19.0-21.4)	21.5 (19.9-23.3)	21.0 (19.4-22.5)	20.0 (18.9-21.2)	21.4 (19.8-23.1)	20.3 (18.8-21.9)	20.0 (18.9-21.1)
	Rich	20.8 (18.9-22.9)	21.7 (19.7-23.9)	20.0 (18.8-21.2)	21.0 (19.4-22.7)	21.6 (19.9-23.3)	20.0 (18.8-21.3)	20.9 (19.3-22.7)	21.0 (19.4-22.8)	20.0 (18.8-21.2)
	Richest	19.8 (17.7-22.0)	20.3 (18.3-22.5)	20.1 (18.7-21.6)	18.0 (16.4-19.8)	19.3 (17.6-21.1)	20.0 (18.5-21.6)	19.3 (17.5-21.2)	19.8 (17.9-21.8)	20.0 (18.6-21.5)

Supplementary Table S2. Prevalence of vitamin B12 and folate deficiency in children and adolescents by socio-demographic and WASH variables.

Characteristics		1-4 years		5-9 years		10-19 years	
		Vitamin B12 deficiency % (95% CI)	Folate deficiency % (95% CI)	Vitamin B12 deficiency % (95% CI)	Folate deficiency % (95% CI)	Vitamin B12 deficiency % (95% CI)	Folate deficiency % (95% CI)
Residence	Urban	14.4[a] (9.9-20.4)	23.5[a] (20.5-26.8)	15.7[a] (11.9-20.4)	31.1[a] (27.4-35.0)	30.5[a] (27.1-34.0)	40.9[a] (35.6-46.3)
	Rural	13.6[a] (11.4-16.3)	22.5[a] (19.5-25.7)	17.8[a] (15.7-20.1)	26.5[a] (23.7-29.5)	31.2[a] (28.2-34.3)	33.8[a] (30.7-37.1)
Currently in school	Yes	-	-	17.4[a] (15.5-19.4)	28.1[a] (25.8-30.5)	31.1[a] (28.3-33.9)	36.7[a] (34.0-39.4)
	No	-	-	16.1[a] (11.7-21.9)	22.5[a] (17.9-27.9)	30.9[a] (26.5-35.6)	33.4[a] (29.4-37.8)
Mother's Schooling	Primary	16.4[a] (12.7-20.9)	25.2[a] (21.4-29.5)	20.4[a] (17.7-23.5)	25.2[a] (22.3-28.4)	26.6[a] (20.3-34.0)	38.8[a] (33.0-44.9)
	Secondary	12.5[ab] (10.6-14.7)	23.9[a] (21.1-26.9)	14.1[b] (12.2-16.2)	30.4[a] (27.5-33.4)	30.8[a] (27.8-34.1)	41.5[a] (38.3-44.7)
	Higher secondary	8.3[b] (5.9-11.5)	16.2[b] (12.9-20.2)	17.1[ab] (13.0-22.2)	32.5[ab] (27.3-38.0)	30.0[a] (20.2-42.2)	41.9[a] (31.0-53.6)
	Graduate and above	16.9[ab] (7.9-32.4)	16.9[ab] (12.6-22.3)	14.4[ab] (9.0-22.3)	23.4[ab] (19.5-27.8)	28.4[a] (22.4-35.3)	34.4[a] (27.8-41.6)
Father's Occupation	Professional	11.6[a] (8.1-16.3)	17.0[a] (13.1-21.8)	17.7[a] (13.6-22.9)	21.1[a] (17.1-25.7)	37.1[a] (28.6-46.5)	23.1[a] (18.7-28.1)
	Sales and services	11.5[a] (8.9-14.8)	22.3[ab] (19.6-25.3)	14.7[a] (11.8-18.3)	30.4[b] (27.4-33.6)	27.9[a] (24.6-31.4)	39.5[b] (35.9-43.1)
	Manual, Agriculture	13.8[a] (11.6-16.3)	25.7[b] (22.3-29.4)	17.5[a] (15.3-20.0)	30.0[b] (27.0-33.1)	31.2[a] (28.5-34.0)	39.3[b] (36.0-42.7)
	Others	20.0[a] (11.7-31.9)	16.5[ac] (12.1-22.0)	20.7[a] (15.5-27.2)	17.1[ac] (13.4-21.5)	31.7[a] (26.7-37.1)	23.6[ac] (19.4-28.3)
Wealth Index	Poorest	13.8[a] (10.2-18.4)	19.9[a] (14.9-26.1)	22.2[a] (17.8-27.3)	20.7[a] (15.4-27.1)	30.7[a] (25.0-37.0)	27.0[a] (21.6-33.2)
	Poor	15.0[a] (10.1-21.6)	23.3[a] (18.2-29.2)	14.7[a] (11.7-18.4)	23.5[a] (20.0-27.4)	28.0[a] (23.2-33.3)	32.0[ab] (27.8-36.6)
	Middle	13.9[a] (10.3-18.5)	25.1[a] (21.3-29.3)	18.0[a] (14.8-21.6)	29.6[ab] (26.0-33.5)	30.0[a] (25.7-34.7)	35.9[ab] (32.1-39.9)
	Rich	13.1[a] (9.7-17.5)	24.4[a] (21.2-28.0)	16.8[a] (14.2-19.9)	33.6[b] (30.4-37.0)	33.5[a] (29.7-37.6)	40.3[b] (36.2-44.6)
	Richest	13.2[a] (9.8-17.6)	20.2[a] (17.4-23.2)	15.0[a] (11.6-19.2)	29.6[ab] (26.6-32.8)	32.9[a] (29.2-36.9)	42.0[b] (37.2-47.0)
Drinking water source	Piped & Improved	13.3[a] (11.2-15.8)	21.0[a] (18.7-23.4)	17.4[a] (15.5-19.4)	26.3[a] (24.0-28.6)	31.1[a] (28.5-33.9)	33.3[a] (30.8-36.0)
	Non-piped & Improved	19.7[a] (10.6-33.6)	32.3[b] (26.4-38.8)	17.9[a] (9.7-30.8)	33.6[ab] (26.3-41.9)	32.5[a] (28.0-37.3)	48.0[b] (40.4-55.6)
	Unimproved	12.1[a] (7.9-18.1)	35.8[ab] (23.0-51.0)	15.9[a] (11.9-21.0)	39.2[b] (30.4-48.8)	29.6[a] (22.2-38.2)	54.7[b] (45.7-63.5)
Hand washing	Basic	13.4[a] (10.4-17.1)	23.0[a] (20.3-25.9)	17.2[a] (15.3-19.3)	28.7[a] (26.3-31.3)	32.7[a] (29.9-35.7)	38.0[a] (35.0-41.1)
	Limited	14.0[a]	26.0[a]	16.3[a]	28.5[ab]	30.6[a]	36.0[ab]

		(11.5-16.9)	(22.3-29.9)	(13.7-19.3)	(24.9-32.4)	(26.6-34.8)	(32.2-39.9)
	No facility	14.7[a] (9.2-22.7)	14.1[b] (10.6-18.5)	20.2[a] (14.5-27.5)	21.3[b] (17.4-25.7)	27.0[a] (22.6-32.0)	28.0[b] (22.7-33.9)
Sanitation	Improved & Not shared	11.9[a] (9.3-15.0)	24.1[a] (21.4-27.0)	16.2[a] (13.5-19.3)	32.2[a] (29.6-34.9)	31.0[a] (28.5-33.7)	39.3[a] (36.3-42.5)
	Improved & Shared	12.9[a] (9.5-17.3)	19.8[a] (16.0-24.3)	13.8[a] (10.9-17.4)	25.8[ab] (22.4-29.5)	27.2[a] (22.5-32.4)	35.3[ab] (30.7-40.0)
	Unimproved	15.9[a] (12.8-19.6)	22.4[a] (18.8-26.5)	19.2[a] (16.4-22.3)	24.4[b] (21.0-28.2)	31.6[a] (27.7-35.8)	31.8[b] (27.9-36.1)

Superscripts [abc] with different letters in the same column indicate estimates with non-overlapping CI

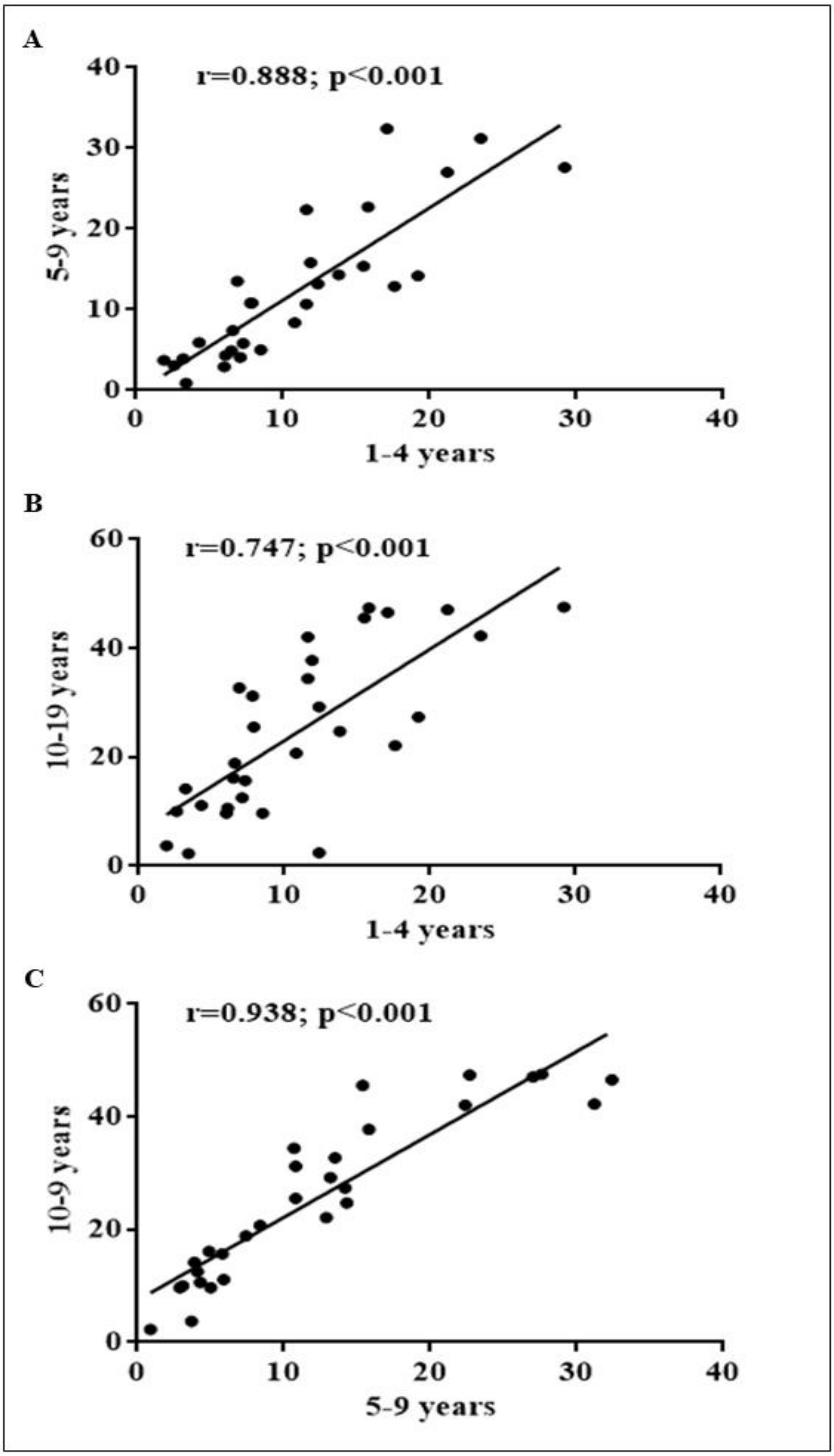

Supplementary Figure S1A. Association between the vitamin B_{12} prevalence by state across all the age groups (A) 1-4 years vs 5-9 years (B) 1-4 years vs 10-19 years and (C) 5-9 years vs 10-19 years.

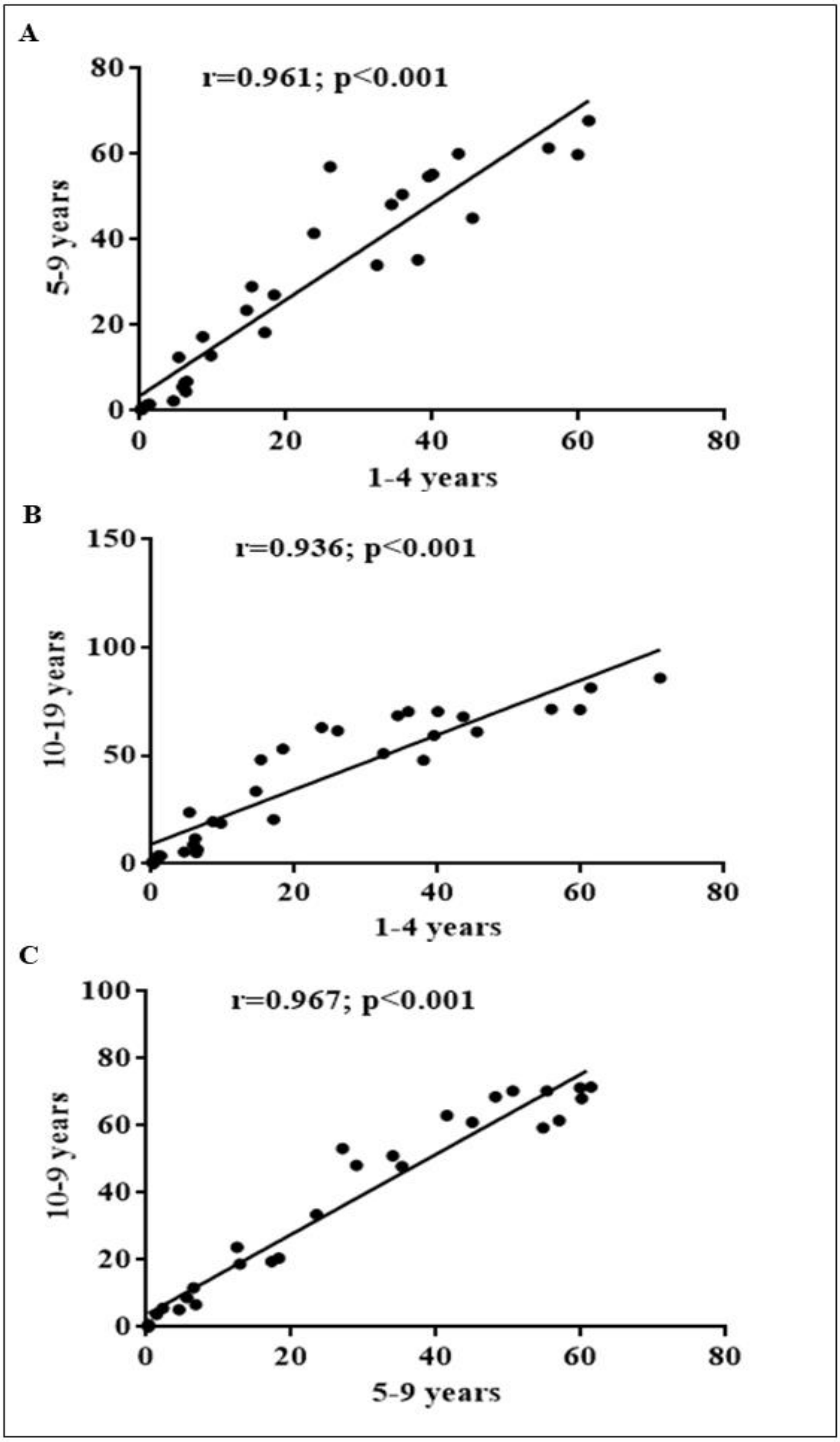

Supplementary Figure S1B. Association between the folate prevalence by state across all the age groups (A) 1-4 years vs 5-9 years (B) 1-4 years vs 10-19 years and (C) 5-9 years vs 10-19 years.
